# Proteome differences associated with fat accumulation in bovine subcutaneous adipose tissues

**DOI:** 10.1186/1477-5956-8-14

**Published:** 2010-03-18

**Authors:** Yong Mei Zhao, Urmila Basu, Michael V Dodson, John A Basarb, Le Luo Guan

**Affiliations:** 1 Department of Agricultural, Food and Nutritional Science, University of Alberta, Edmonton, Alberta T6G 2P5, Canada; 2 Department of Life Science, Xi'an University of Arts and Science, Shaanxi, Xi'an710065, PR China; 3 Department of Animal Sciences, Washington State University, PO Box 646310, Pullman, Washington, 99164, USA; 4 Alberta Agriculture and Rural Development, Lacombe Research Centre, Lacombe, AB, T4L1W1, Canada

## Abstract

**Background:**

The fat components of red meat products have been of interest to researchers due to the health aspects of excess fat consumption by humans. We hypothesized that differences in protein expression have an impact on adipose tissue formation during beef cattle development and growth. Therefore, in this study we evaluated the differences in the discernable proteome of subcutaneous adipose tissues of 35 beef crossbred steers [Charolais × Red Angus (CHAR) (n = 13) and Hereford × Angus (HEAN) (n = 22)] with different back fat (BF) thicknesses. The goal was to identify specific protein markers that could be associated with adipose tissue formation in beef cows.

**Results:**

Approximately 541-580 protein spots were detected and compared in each crossbred group, and 33 and 36 protein spots showed expression differences between tissues with high and low BF thicknesses from HEAN and CHAR crossbed, respectively. The annexin 1 protein was highly expressed in both crossbred steers that had a higher BF thickness (p < 0.05) and this was further validated by a western blot analysis. In 13 tissues of CHAR animals and 22 tissues of HEAN animals, the relative expression of annexin 1 was significantly different (p < 0.05) between tissues with high and low BF thicknesses.

**Conclusion:**

The increased expression of annexin 1 protein has been found to be associated with higher BF thickness in both crossbred steers. This result lays the foundation for future studies to develop the protein marker for assessing animals with different BF thickness.

## Background

The adipose tissue content of meat products not only has an impact on the economic value for producers, but it also impacts the nutrition and health of red meat consumers. For beef production, it is desirable to produce beef cattle with a moderate amount of adipose tissue in the correct adipose depot (marbling fat) to have carcasses with an acceptable economic value. However, adipose tissue formation in beef cattle is a complicated biological process associated with the genetic background, development, and nutrition of an animal, maintained by unique molecular signaling pathways [1-3]. Gene expression analyses, using a novel *in vitro *model of cattle adipocytes [4-6], showed that genes for peroxisome proliferator-activated receptors (PPARγ), CCAAT-enhancer binding proteins (C/EBPα, C/EBPβ) and sterol regulatory element binding protein (SREBP 1c) are directly or indirectly involved in the regulation of bovine adipogenesis [7-9]. In addition, the Wdnm1-like protein, a distant member of the whey acidic protein/four-disulfide core family, was shown to be associated with adipogenesis in livestock species as a remodeler of the extracellular milieu in adipogenesis and/or as a differentiation-dependent gene in white and brown adipogenesis [10]. In contrast to previous studies, and through the use of other cell models, many other genes have been found to be up- or down-regulated during the early stage of adipocyte differentiation [11-14].

The association of adipose tissue protein profiles in beef cows with the exhibition of different production traits remains unknown. Moreover, the gene expression levels do not always correspond to the protein levels. The meat composition of the animal can be directly associated with the end products of gene expression only if there is a synthesis of functional/viable proteins. Biologically active proteins can be modified by the efficiency of translation, by post-translational modifications, and by the rate and extent of proteolysis, for instance. Therefore, it is necessary to combine information on the expression of both the genes and proteins to create a complete picture of bovine adipogenesis [15]. Two-dimensional gel electrophoresis (2-DE) and mass spectrometry (MS) are methods that are widely used to investigate the physiologically relevant proteins associated with various biochemical and physiological changes in development, growth or metabolism of skeletal muscle and associative adipogenesis [16,17].

Proteome changes are associated with the complex mechanisms of postmortem processes that occur during the conversion of muscle to meat. Proteolysis, changes in intracellular pH, ion transport and water holding capacity [18,19] are variables that have been linked to meat tenderness. The adipose tissue components are also altered during the conversion of muscle to meat, and proteins involved in lipogenesis, glycolysis, lipolysis, fatty acid oxidation, and energy transfer are down-regulated, while numerous growth enzymes are actually up-regulated in intramuscular adipocytes in postmortem porcine adipocytes [20]. Moreover, adipocyte fatty acid-binding protein expression at both the mRNA and protein levels have been used as indicators of intramuscular adipocyte number, and hence fat turnover [21].

There has still been no characterization of the proteome changes associated with back fat (BF) thickness in beef cattle. The objectives of this study were to perform a comparative proteomic analysis from subcutaneous adipose tissues of beef steers with high and low BF thickness, and to identify the major protein markers associated with fat metabolism in beef cattle. In order to conduct this study, we selected animals that were physically divergent, but similar in age and developmental stage. Moreover, we used adipose tissue from only the largest adipose depot (BF; subcutaneous adipose depot) because it is economically inefficient, and it is metabolically different from other adipose tissue depots in cattle and pigs [5,22]. Previously, using microarray analysis, we found that 360 genes were differentially expressed in animals with higher and lower BF thickness, and that the association between gene expression and BF thickness was somehow different in CHAR and HEAN animals [9]. Here, we further analyzed the pattern of differential protein expression in BF tissues from these animals.

## Results

### Comparison of the BF thickness and other carcass trait in two crossbred animals

The differences in BF thickness between the HEAN (n = 22) and CHAR (n = 13) steers, and other characteristics, including the age at slaughter, the slaughter mass, the cutability, the rib eye area and the marbling score, are shown in Table 1. For both the breeds, the BF was approximately 2 times higher in the high BF group than that in the low BF group (P < 0.0001). Table 2 provides the data on BF thickness and other characteristics based on 6 animals per racial group (High BF, n = 3; Low BF, n = 3) used for comparative proteomics analysis in this study. Again in both crossbred groups, the BF was approximately 2 times higher in the high BF group than that in the low BF group (P = 0.025 in CHAR crossbred, P = 0.002 in HEAN crossbred). All of the other variables that were measured in the BF groups were not different (P > 0.05).

**Table 1:** Comparison of carcass traits showing the back fat classification and assessment of age effect within the same breed (CHAR and HEAN) and variance between the two breeds

	CHAR (mean ± SE) (n = 13)			HEAN (mean ± SE) (n = 22)			Variance between two breeds
	**LOW BF**	**High BF**	**Significance (P value)**	**LOW BF**	**High BF**	**Significance (P value)**	**Significance** **(P value)**

Age at slaughter (d)	466.50 ± 6.76	467.00 ± 8.22	0.95	482.00 ± 7.29	475.00 ± 4.77	0.39	< 0.0001
Slaughter mass (kg)	559.12 ± 40.13	558.12 ± 25.71	0.94	556.08 ± 11.84	550.42 ± 19.83	0.34	0.91
BF thickness (mm)	5.00 ± 0.41	11.25 ± 0.75	< 0.0001	9.00 ± 0.45	15.00 ± 0.52	< 0.0001	< 0.0001
Cutability (%)	62.75 ± 0.75	56.50 ± 0.87	< 0.0001	58.50 ± 0.62	53.17 ± 0.31	< 0.0001	< 0.0001
Rib eye area (cm^2^)	93.00 ± 9.19	79.75 ± 5.85	0.17	78.33 ± 3.53	74.33 ± 3.50	0.75	0.0139
Marbling score	452.50 ± 17.01	452.50 ± 21.36	0.81	436.67 ± 23.76	503.33 ± 5.58	0.04	0.3116

General Linear Model (GLM) procedure from SAS system was used to evaluate the fixed effects of racial group (CHAR and HEAN), and back fat group (High BF and Low BF, nested within racial group) on carcass traits.

**Table 2:** The characteristics of CHAR and HEAN steers selected for proteomic analysis

	CHAR (mean ± SE)			HEAN (mean ± SE)		
	**LOW BF** **(n = 3)**	**High BF** **(n = 3)**	**Significance (P value)**	**LOW BF** **(n = 3)**	**High BF (n = 3)**	**Significance (P value)**

Age at slaughter (d)	470.6 ± 8.83	470.00 ± 10.81	1.000	480.3 ± 12.44	470.00 ± 5.85	0.681
Slaughter mass (kg)	513.7 ± 57.35	535.0 ± 15.87	0.683	557.0 ± 16.5	550.00 ± 38.4	0.681
BF thickness (mm)	4.50 ± 0.50	11.66 ± 0.88	0.025	8.33 ± 0.66	16.00 ± 0.57	0.002
Cutability (%)	63.00 ± 1.00	55.67 ± 0.33	0.132	59.00 ± 0.57	53.00 ± 0.57	0.141
Rib eye area (cm^2^)	90.33 ± 12.44	74.00 ± 1.53	0.681	79.00 ± 7.09	72.66 ± 6.48	0.536
Marbling score	456.67 ± 23.33	446.67 ± 29.06	1.000	430.00 ± 40.41	500.00 ± 11.5	0.423

### Proteome profiling of subcutaneous adipose tissues with different BF thicknesses

**Figure 1:**
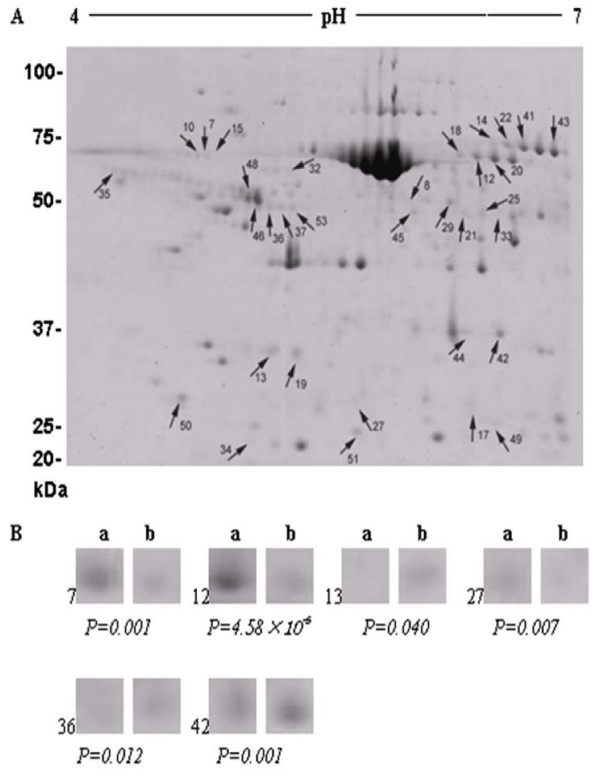
**Two-dimensional gel images of differentially expressed proteins with a high or low BF thickness in CHAR steers (A)**. The arrows indicate significantly differentially expressed proteins (spots) (P < 0.05) after identification by matching, background subtraction and spot volume normalization using the Progenesis software. The selected protein spots for MS analysis are also shown (B), demonstrating changes in intensity and significant difference.

**Figure 2:**
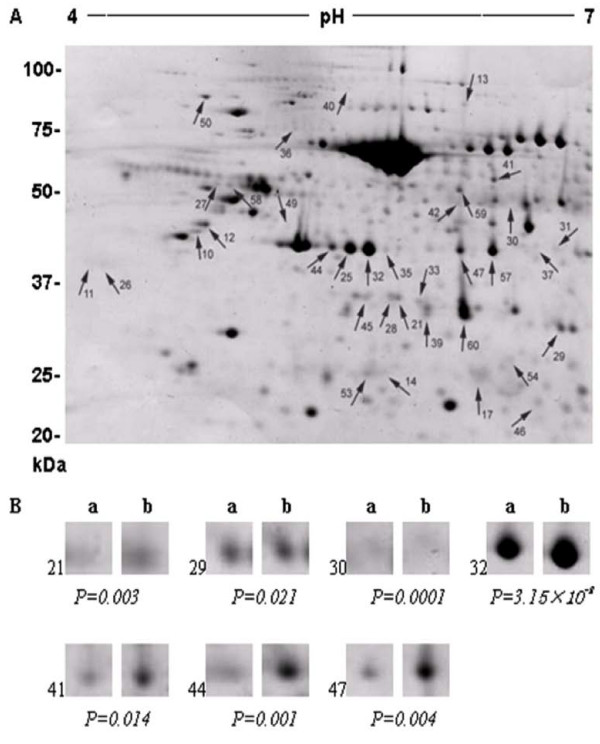
**Two-dimensional gel images of differentially expressed proteins from HEAN steers with a high or low BF thickness (A)**. The arrows indicate significantly differentially expressed proteins (spots) (P < 0.05) after identification by matching, background subtraction and spot volume normalization using the Progenesis software. The selected protein spots for MS analysis are also shown (B), demonstrating changes in intensity and significant difference.

Differences in the protein profiles were found to be associated with the BF thickness in both HEAN and CHAR crossbred animals. In total, a range of 541-580 spots were resolved on each replicate gel from proteins extracted from animals with a high or low BF thickness and from different crossbred groups (Figures 1 &2). From CHAR animal adipose tissues, a total of 33 proteins were significantly altered, with 15 proteins up-regulated and 18 proteins down-regulated in the low BF group compared to the high BF group. The difference in expression levels ranged from 1.4-3.7 fold (P < 0.05). From HEAN animal adipose tissues, a total of 36 proteins were significantly different, with 24 proteins up-regulated and 12 proteins down-regulated in the low BF group compared to the high BF group. The difference in expression levels ranged from 1.3-2.0 fold (P < 0.05).

### Characterization of differentially expressed proteins

After normalization, volume calculation, and statistical analysis with the Progenesis Samespots software, 12 and 10 spots from each crossbred group were chosen for Mass Spectrometry/Mass Spectrometry (MS/MS) analysis, and 6 and 7 proteins, respectively, were characterized. Table 3 shows the characteristics of the identified proteins, including the number of peptides matched, the percent coverage, the isoelectric points (pI), the molecular weight, the accession number, the score, the matched sequence and the fold change. A single peptide identity was obtained for 11 out of 13 spots that were sequenced. However, for spots H29 and H44, two peptide identities were obtained (Table 3). This might be due to the very close molecular mass and/or pI of these peptides, making the unambiguous identification of these spots impossible [23].

**Table 3:** Identified proteins in HEAN and CHAR steers by 2D-gel and MS analysis

Spot^a^	Accession^b^	Protein name	Score^c^	C^d^	N^e^	MW(kDa/pI)	sequence^f^	Average Fold change^g^
C7	114053019	alpha-1-B glycoprotein [Bos taurus]	255/56	17	7	54.1/5.29	R. FPLGPVTSTTR.G R.LEGEDQFLEVAEAPEATQATFPVHR.A R.VLSPAGPEAQFELR.G R.ALWTGALTPGR.D R.CEAEVPDVSFLLLR.A R.SLLSELSDPVELR.V	-3.7
C12	2501351	serotransferrin precursor [Bos taurus]	176/55	7	3	79.9/6.75	K.SVDDYQECYLAMVPSHAVVAR.T K.GEADAMSLDGGYLYIAGK.C K.FDEFFSAGCAPGSPR.N	-2.8
C13	3024627	Alpha-soluble NSF attachment protein [Bos taurus]	289/54	32	8	33.6/5.39	K.NSQSFFSGLFGGSSK.I K.HDAATCFVDAGNAFK.K K.ADPQEAINCLMR.A K.VAGYAAQLEQYQK.A K.YEELFPAFSDSR.E K.LLEAHEEQNVDSYTEAVK.E R.LDQWLTTMLLR.I	+2.7
C27	119924886	purine nucleoside phosphorylase [Bos taurus]	423/56	39	10	32.4/6.25	M.ENGFTYEDYQDTAK.W R.LTQAQTFDYSEIPNFPKSTVPGHAGR R.DHINLPGFSGENPLR.G R.FPAMSDAYDR.D R.NLGADAVGMSTVPEVIVAR.H R.VFGFSLITNK.V K.VIMDYESQGK.A K.ADHKEVLEAGK.Q	-1.6
C36	162287374	polymerase I and transcript release factor isoform 2 [Bos taurus]	311/57	20	9	43.4/5.49	K.SDQVNGVLVLSLLDK.I K.IIGAVDQIQLTQAQLEER.Q R.QAEMEGAVQSIQGELSK.L K.KLEVNEAELLR.R K.LEVNEAELLR.R K.ATEMVEVGAEEEEGGAER.G	+1.8
C42	74	annexin I [Bos taurus]	600/57	45	20	39.2/6.44	K.QAWFIENEEQEYIK.T K.GGPGSAVSPYPTFNPSSDVEALHK.A K.GVDEATIIEILTK.R K.KALLGHLEEVVLALLK.T K.ALLGHLEEVVLALLK.T K.TPAQFDAEELR.A K.GLGTDEDTLNEILASR.T R.SEELAVNDDLADSDAR.A K.GTDVNVFTTILTTR.S K.VLDLELK.G K.CATSQPMFFAEK.L K.LYGISLCQAILDETK.G	+1.7
H21	164420731	Transaldolase 1 [Bos taurus]	159/56	10	3	37.8/7.03	K.LGGSQEEQITNAIDK.L K.LFVLFGAEILK.K K.SYEPQEDPGVK.S	+1.7
H29	27807289 78365297	Annexin [Bos taurus] glycerol-3-phosphate dehydrogenase 1 (soluble) [Bos taurus]	494/56 370/56	28 29	11 10	38.8/6.92 38.2/6.42	R.DALNIETAIK.T K.GVDEVTIVNILTNR.S K.TPAQYDASELK.A R.TNQELQEINR.V K.DIVSDTSGDFR.K R.AEDGSVIDYELIDQDAR.D K.SYSPYDMLESIK.K K.SLYYYIQQDTK.G K.VCIVGSGNWGSAIAK.I R.VTMWVFEEDIGGR.K R.KLTEIINTQHENVK.Y K.LTEIINTQHENVK.Y K.ADTIGVSLIK.G K.LISEVIGER.L R.LGIPMSVLMGANIANEVADEK.F R.LGLMEMIAFAK.L K.FPLFTAVYK.V	+1.5
H30	194680257	glucose-6-phosphate dehydrogenase [Bos taurus]	270/56	16	7	62.9/6.9	R.DGLLPEDTYIVGYAR.S R.NSYVAGQYDDTASYK.R R.DLQSSNQLSNHIASLFHEDQIYR.I K.KPGMFFNPEESELDLTYGNR.Y K.LPDAYER.L R.GPVEADELMK.R	-1.5
H32	62751863	brain creatine kinase [Bos taurus]	784/56	46	27	42.9/5.47	R.FPAEDEFPDLSGHNNHMAK.V K.VLTPELYAELR.A R.GFCLPPHCSR.G K.LAVEALSSLDGDLAGR.Y K.SMTEAEQQQLIDDHFLFDKPVSPLLLASGMAR K.PVSPLLLASGMAR.D K.TFLVWINEEDHLR.V R.FCNGLTQIETLFK.S K.RGTGGVDTAAVGGVFDVSNADR.L R.LGFSEVELVQMVVDGVK.L K.LLIEMEQR.L R.LEQGQAIDDLMPAQK.-	+1.5
H41	221325670 115496400	malic enzyme 1, NADP(+)-dependent, cytosolic [Bos taurus] dihydropyrimidinase-like 2 [Bos taurus]	618/56 356/56	26 16	16 6	63.9/5.86 62.6/5.95	R.DLAFTLEER.Q R.QQLNIHGLLPPSFISQDVQVLR.V R.LNSDFDR.Y R.YLLLMDLQDR.N K.VLMSDIEK.F R.GLFISIHDR.G R.GHISSVLNAWPEDVIK.A K.AIVVTDGER.I K.IWLVDSK.G K.AECTAEQCYK.L R.AIFASGSPFDPVTLPSGK.S K.IFLTTAEVIAQQVSDK.H K.TATVYPEPPNK.E K.QIGENLIVPGGVK.T R.DIGAIAQVHAENGDIIAEEQQR.I R.SITIANQTNCPLYITK.V K.DNFTLIPEGTNGTEER.M R.GSPLVVISQGK.I R.GLYDGPVCEVSVTPK.T	+1.4
H44	13096153	Retinal Creatine Kinase [Bos taurus]	784/56	46	27	42.8/5.48	R.FPAEDEFPDLSGHNNHMAK.V K.VLTPELYAELR.A R.GFCLPPHCSR.G K.LAVEALSSLDGDLAGR.Y K.SMTEAEQQQLIDDHFLFDKPVSPLLLASGMAR.D K.PVSPLLLASGMAR.D K.TFLVWINEEDHLR.V R.FCNGLTQIETLFK.S K.RGTGGVDTAAVGGVFDVSNADR.L R.LGFSEVELVQMVVDGVK.L K.LLIEMEQR.L R.LEQGQAIDDLMPAQK.-	+1.6
H47	75832090	isocitrate dehydrogenase 1 (NADP+), soluble	1022/56	55	36	47.1/6.13	K.IQGGSVVEMQGDEMTR.I K.CATITPDEK.R R.NILGGTVFR.E R.LVSGWVKPIIIGR.H R.ATDFVVPGPGK.V K.VEISYTPSDGSPK.T K.TVYLVHNFTESGGVAMGMYNQDK.S K.SIEDFAHSSFQMALSK.N R.FKDIFQEIYDK.Q K.SEFEAQNIWYEHR.L R.LIDDMVAQAMK.S K.SEGGFIWACK.N K.TVEAEAAHGTVTR.H K.GQETSTNPIASIFAWTR.G K.ALEEVCIETIEAGFMTK.D K.DLAACIK.G R.SDYLNTFEFMDK.L K.LGENLQLK.L	+1.4

^a^Spot number as given on the 2-D gel image. C spot from CHAR crossbred, H spot from HEAN crossbred

^b^gi number from NCBI database of matched protein

^c^Mascot score/Mascot threshold score for each identified protein.

^d^Percent sequence coverage (%)

^e^Number of matched peptides

^f^The sequence of matched peptides

^g^The expression level (in fold change) in High BF tissues compared to Low BF tissues

**Figure 3:**
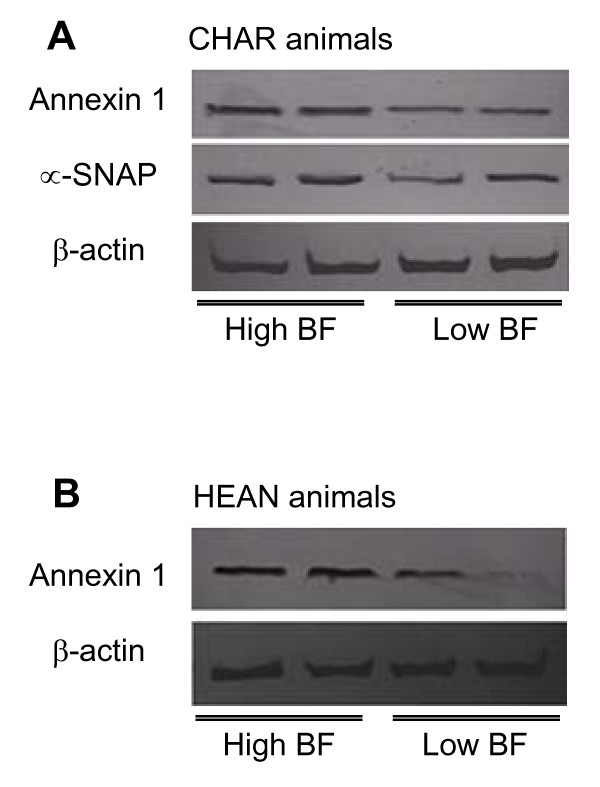
**Western blot analysis of annexin 1 and α-SNAP expression in adipose tissues of CHAR steers (A), and annexin 1 expression in adipose tissues of HEAN steers (B)**. β-actin was used as an internal standard in this study.

All 13 proteins identified in this study were categorized into different cellular functional groups based on the available literature and the protein databases Pfam http://pfam.sanger.ac.uk or InterPro http://www.ebi.ac.uk/interpro[24]. Different functional proteins were found in the different crossbred animals. For the CHAR animals, proteins involved in cell immunity (immunoglobulin, spot C7) and protein synthesis (purine nucleoside phosphorylase, spot C27) were significantly down-regulated with increasing levels of BF thickness. Proteins related to the cell cycle (polymerase I and transcript release factor isoform 2, spot C36) were up-regulated with an increase in BF thickness. Three proteins (spots C12, C13 and C42) associated with a function in cellular trafficking, annexin 1, the alpha-soluble N-ethyl-maleimide-sensitive fusion protein (NSF) attachment protein (α-SNAP) were up-regulated and the serotransferrin precursor, was down-regulated with high and low BF thickness, respectively (Table 3). For HEAN animals, proteins associated with energy metabolism (brain creatine kinase, spot H32, and retinal creatine kinase, spot H41) were up-regulated with an increase in BF thickness. Moreover, a number of proteins function in the oxidation-reduction process and carbon metabolism, including protein spots H21, H29, H30, H41 and H47. Another protein, glucose-6-phosphate dehydrogenase (G6PDH, spot H30) was down-regulated in tissues with increased BF thickness. To further confirm whether expression of the differentially expressed proteins was associated with BF thickness, we utilized western blotting. An annexin 1 antibody was used to examine the annexin-1 expression profile in adipose tissues from 13 CHAR animals and 22 HEAN animals, while antibody to α-SNAP was used to examine α-SNAP levels in adipose tissues from 13 CHAR animals. Annexin 1 expression showed a positive association with increased BF thickness as annexin 1 had a lower expression profile in low BF animals when compared to high BF animals in both crossbreds (p < 0.05, Table 4, Figure 3), while the α-SNAP protein level was altered with a change in BF thickness in the HEAN animals and no change was observed in CHAR animals (Figure 3).

**Table 4:** Comparison of annexin 1 protein expression in adipose tissues from CHAR (n = 13) and HEAN (n = 22) crossbred steers using western blot analysis

Animal	Proteins	Mean ± SE (arbitrary units)		Significance (p value)
		High BF	Low BF	
CHAR	Annexin 1	1.02 ± 0.12	0.66 ± 0.07	0.04
HEAN	Annexin 1	1.08 ± 0.18	0.52 ± 0.16	0.03

Shown are the means ± SE of densitometry results (arbitrary units) normalized to actin protein expressions.

## Discussion

Back fat thickness is one of the major quantitative traits that has an impact on carcass and meat quality in beef cattle and in combination with marbling can be used for stratification of the beef carcasses [25]. The proteome changes that are associated with BF thickness in beef cattle have not yet been well characterized, thus we performed a comparative proteomic analysis of subcutaneous adipose tissues from beef steers with a high and low BF thickness, in order to identify major protein markers associated with fat metabolism in beef cattle.

As shown in Figure 1B and Figure 2B, the identified proteins were significantly (p < 0.05) affected by BF thickness and the host animal. A number of proteins, such as those involved in cellular trafficking (annexin 1, α-SNAP and serotransferrin precursor), were differentially expressed in the CHAR animals, although the genes encoding these proteins had not been previously identified in microarray studies. α-SNAP belongs to the soluble NSF attachment protein receptor (SNARE) protein complex, which are a group of membrane proteins associated with distinct membrane compartments of the secretory and endocytic trafficking pathways, and contribute to the specificity of membrane fusion [26]. These proteins have been shown to function at all sites of constitutive and regulated secretion in eukaryotes [27]. Members of the SNARE complex include two types of cytosolic proteins, NSF and the SNAPs (α-, β- and γ-soluble NSF attachment proteins). Two-dimensional gel analysis of six CHAR animals demonstrated that there is an increased expression of α-SNAP with increased BF thickness in these animals (Table 3), suggesting that membrane secretion plays a role in fat accumulation and might cause the difference in BF thickness. However, the expression of α-SNAP was not significantly higher when a larger numbers of animals (n = 13) were compared (data not shown). Therefore, the function of α-SNAP in fat depot formation needs further validation in CHAR animals.

From both animal treatment groups, annexin 1 was detected in all animals, despite the BF thickness, although it was more highly expressed in the adipose tissues with a higher BF thickness (Table 4). The annexins (or lipocortins) are a family of proteins that are ubiquitously distributed in different tissues and cell types of higher and lower eukaryotes, including mammals, fish, and birds [28]. This protein functions as calcium-dependent phospholipid-binding protein [29]. A recent study by Braun et al. [30] reported that the annexin family is linked to an inhibition of phospholipase activity, exocytosis and endoctyosis, signal transduction, organization of the extracellular matrix, resistance to reactive oxygen species and DNA replication. Annexin 1 is the first member of the annexin family of proteins that has been characterized to have the ability to bind (i.e. to annex) to cellular membranes in a calcium-dependent manner [31]. The down-regulation of annexin 1 in animals with a higher BF thickness suggests that the cell membrane-binding function of annexin 1 might be important in fat depot formation. This is the first study to report the association of increased annexin 1 expression with increased BF thickness in beef cattle. Further study to identify the role of annexin 1 in adipogenesis will be performed in adipocyte cell lines.

Several proteins in HEAN animals were differentially expressed and associated with differences in BF thickness. A large number of these proteins function in the oxidation-reduction process and carbon metabolism for the transport of energy. For example, glycerol-3-phosphate dehydrogenase (G3PDH), Malic enzyme 1, and isocitrate dehydrogenase were over-expressed in tissues with high BF. Glycerol-3-phosphate dehydrogenase is a marker of triglyceride synthesis, while the other two enzymes are involved in glycerol degradation via the glycerol kinase pathway and catalyze the oxidative decarboxylation of malate to form pyruvate suggesting that these proteins might play major roles in the fat metabolic pathways. Malic enzyme [(*S*)-malate:NAD(P)^+ ^oxidoreductase (decarboxylating); EC 1.1.1.38-40] catalyzes the oxidative decarboxylation of malate to pyruvate and carbon dioxide, using NAD^+ ^or NADP^+ ^as the electron acceptor [32-34]. The NADP^+^-dependent isocitrate dehydrogenase IDH (EC 1.1.1.42) which is found in both mitochondria (IDH2) and cytosol (IDH1), catalyzes the decarboxylation of isocitrate to α-ketoglutarate (α-KG) and generates NADPH, which is a primary source of reducing equivalents utilized for fatty acid synthesis in bovine [35,36].

Host genetics play a role in the differences in carcass traits. The crossbred HEAN have the genetic components of Hereford and Angus, while the CHAR animals have the genetic components of Charolais. Thus, it is not surprising that different proteins were detected for the two animal groups because Hereford and Angus breeds mature and fatten earlier than Charolais [37]. Therefore, the differences in protein expression might point to particular proteins that control fat formation in these two groups of animals. Upon further investigation, the unanalyzed spots might reveal additional proteins that help determine the variation in adipogenesis for the HEAN and CHAR beef cows.

In conclusion, a comparative proteomic analysis of subcutaneous adipose tissues of beef steers with a high and low BF thickness identified a major protein marker (annexin 1) associated with fat accumulation and metabolism. Considering that this adipose depot is the largest and the most energetically inefficient, protein markers such as annexin 1 might be an indicator of BF accumulation, however, for this, annexin 1 levels will have to be determined in young animals in future studies. Furthermore, expression of a protein marker in combination with ultrasound measurement of BF might provide an additional assessment criterion to reduce the impact of the subcutaneous adipose depot on red meat animal production. The protein markers and their associations with the genetic makers could potentially identify animals producing less BF. Further work is in progress to identify proteins that have a consistent differential expression in fat metabolic pathways, and to define the role(s) of annexin 1 in the depot-specific processes of adipogenesis and lipid metabolism.

## Methods

### Animal sampling

Twenty-two Hereford × Aberdeen Angus (HEAN) and thirteen Charolais × Red Angus (CHAR) crossbred steers were fed and slaughtered at the Lacombe Research Centre in Alberta, Canada. The details of feeding and management were followed, as outlined by Basarab et al [38]. The steers were raised following the guidelines of the Canadian Council on Animal care. The steers were harvested at the Lacombe Research Centre abattoir when the average group ultrasound BF thickness reached 8 to 9 mm. After processing, the weights of the right and left halves of the warm carcass, the cold carcass weight, the BF thickness at the 12^th ^rib, the longissimus thoracis area at the 12 - 13^th ^rib position (REA), the estimated cutability, and the marbling score were recorded. Differences in the carcass characteristics were compared and summarized in Table 1. The subcutaneous adipose tissues were collected immediately after the animals were slaughtered, placed into liquid nitrogen, and stored at -80°C until further analysis.

### Protein extraction

Proteins were extracted from adipose tissues (100 mg each) by grinding in 0.3 mL lysis buffer (40 mM Tris-HCl, pH 8.0, 20 mM DTT, 0.5 mM PMSF containing 1% Triton TM-100) for 10 min at room temperature. The lysates were centrifuged at 13,000 rpm for 10 min. The protein concentration in the supernatant was determined with the Bradford method [39]. In total, proteins from six (high BF n = 3; low BF n = 3) adipose tissues from the HEAN and six (high BF n = 3; low BF n = 3) adipose tissues from the CHAR steers were analyzed by two-dimensional gel electrophoresis analysis.

### Two-dimensional gel electrophoresis

Three protein extractions were performed on each of the tissue samples, for a total of 9 independent protein samples from each BF group, and 18 samples from each crossbred group (3 high BF and 3 low BF samples from HEAN and 3 high BF and 3 low BF from CHAR). Isoelectric focusing (IEF) was performed following the procedures as described by Lee et al [39]. Briefly, the Immobiline™ DryStrip gels (IPG strips) (7 cm, pH 4-7, Amersham Biosciences, Quebec, Canada) were rehydrated with 60 μg of protein in 125 μl of the solubilization solution (8 M urea, 2% CHAPS, 1% IPG buffer (pH 4-7 NL), 13 mM DTT, and a trace of bromophenol blue). The IPG-strips were rehydrated with the sample at room temp for 12 hr. IEF was conducted using IPGphor IEF system (Amersham Bioscience) for 0.5 h at 100 V, 0.5 h at 250 V, a 1.5 h gradient to 4000 V, 1.5 h at 4000 V, resulting in a total of 10000 V/h. The gel strips were equilibrated in two steps for 10 min each with gentle agitation. The first equilibration solution contained 50 mM of pH 8.8 Tris-HCl buffer with 6 M urea, 30% glycerol, 2% SDS, and 1% DTT to ensure that any disulfide bonds were reduced. In the second equilibration solution, DTT was replaced with 2.5% iodoacetamide (IAA) to alkylate the proteins. After equilibration, the IPG strips were rinsed gently with water, and were blotted to remove excess equilibration buffer, then applied onto 10% SDS-PAGE gels and overlaid with a solution of 0.5% agarose with a trace of bromophenol blue. The gel was run at 50 V for 1 h followed by 100 V for 1.5 h, using the mini gel system (Bio-Rad, Ontario, Canada). The proteins were visualized with Coomassie Brilliant Blue R-250.

### Protein identification

The gel images were scanned with Imagescanner (Amersham Biosciences). For each gel, the relative abundance of the resolved protein spots was quantified and data within each spot was normalized by volume calculation, using the Progenesis Samespots software (Nonlinear Dynamics, North Carolina, USA). The spots that were significantly different (p < 0.05) with reproducible changes (up- or down-regulation) in all of the replicates were selected for further analysis. The spots were selected based on the fold change in expression of the proteins, as well as their intensity and location on the gels. The protein spots were then excised from the gel and analyzed for sequential mass analysis measurements using ion trap tandem mass spectrometry (Ion trap MS/MS) at the Institute for Biomolecular Design, University of Alberta. Data was provided for peptides with a charge state of two or three. All of the data was processed using the Mascot (Matrix Science Inc., Boston, MA) and the NCBI non-redundant protein database.

### Western blot analysis

Western blot analysis was performed as described by Jiang et al [40]. Thirty μg of protein were separated using a 10% SDS-PAGE at 50 V for 0.5 h, followed by 100 V for 1.5 h, and were then electrotransferred to a PVDF membrane (Bio-Rad Immobilon-P) in transfer buffer (50 mM Tris, 190 mM Glycine, and 20% Methanol) at 100 V for 100 min at 4°C with constant stirring. The membranes were blocked overnight at 4°C in TBS containing 5% w/v nonfat dry milk, before incubation with primary antibody followed by HRP-conjugated secondary antibody containing TBS-0.5% Tween-20, nonfat dry milk (5% w/v), and BSA (1-3%). To detect the annexin 1 protein, the blots were incubated with an anti-annexin 1 antibody (sc-12740, Santa Cruz Biotechnology, Santa Cruz, CA, USA) at a dilution of 1:500. To detect the α-SNAP protein, the blots were incubated with an anti- α-SNAP antibody (sc-58217, Santa Cruz Biotechnology) at a dilution of 1:500. The secondary goat anti-mouse IgG-HRP (sc-2005, Santa Cruz Biotechnology) was used at a dilution of 1:5000 and the blots were incubated at room temperature for 60 min. Similarly, to detect the internal standard (β-actin), the blots were incubated with a primary rabbit polyclonal antibody to beta-actin (ab8227, abcam, Massachusetts, USA) at a dilution of 1:3000. The secondary goat polyclonal to rabbit IgG-H&L (ab6721, abcam) was used at a dilution of 1:3000, and the blots were incubated at room temperature for 60 min. The blots were developed using a 3,3',5,5'-tetramentylbenzidine substrate kit. The background-corrected signal intensity for each spot was normalized, and the band densities in the Western blots were measured using the AlphaEase software for Windows (Alpha Innotech, Canada). Protein expression was described as the relative intensity of the target protein/β-actin ratio.

### Statistical analysis

Statistical analyses of proteome level changes were performed using the SAS System version 9.2 (SAS Institute, Cary, NC, USA). General Linear Model (GLM) procedure from SAS system was used to evaluate the fixed effects of racial group (CHAR and HEAN), and back fat group (High BF and Low BF, nested within racial group) on carcass traits. All of the data is expressed as the Mean ± standard error (SE) and differences at p < 0.05 were considered significant.

## List of abbreviations

CHAR: Charolais × Red Angus; HEAN: Hereford × Angus; BF: Back fat; 2-DE: two-dimensional gel electrophoresis; MS: mass spectrometry; NSF: N-ethyl-maleimide-sensitive fusion protein; α-SNAP: alpha-soluble NSF attachment protein; G3PDH: glycerol-3-phosphate dehydrogenase; IEF: isoelectric focusing; IPG strips: Immobilized pH gradient strips; SE: standard error.

## Competing interests

The authors declare that they have no competing interests.

## Authors' contributions

LLG, YMZ and UB designed the experiments and YMZ performed the 2D-gel electrophoresis and analysis, and the draft manuscript writing. UB and LLG supervised the experiments and the data analysis. MVD contributed to the data interpretation and the original manuscript writing. JAB performed the animal experiments and supplied all of the carcass data. UB, MVD and LLG finalized the manuscript.

All authors have read and approved the final manuscript.
